# Prediction of Wellness Condition for Community-Dwelling Elderly via ECG Signals Data-Based Feature Construction and Modeling

**DOI:** 10.3390/ijerph191711136

**Published:** 2022-09-05

**Authors:** Yang Zhao, Fan Xu, Xiaomao Fan, Hailiang Wang, Kwok-Leung Tsui, Yurong Guan

**Affiliations:** 1 School of Public Health (Shenzhen), Sun Yat-sen University, Shenzhen 518000, China; 2 School of Mechanical and Electronic Engineering, Wuhan University of Technology, Wuhan 430070, China; 3 College of Big Data and Internet, Shenzhen Technology University, Shenzhen 518000, China; 4 School of Design, The Hong Kong Polytechnic University, Hong Kong, China; 5 Grado Department of Industrial and Systems Engineering, Virginia Polytechnic Institute and State University, Blacksburg, VA 24061, USA; 6 Department of Computer Science, Huanggang Normal University, Huanggang 438000, China

**Keywords:** electrocardiogram, elderly care, wellness, feature construction, predictive models, machine learning methods

## Abstract

The accelerated growth of elderly populations in many countries and regions worldwide is creating a major burden to the healthcare system. Intelligent approaches for continuous health monitoring have the potential to promote the transition to more proactive and affordable healthcare. Electrocardiograms (ECGs), collected from portable devices, with noninvasive and cost-effective merits, have been widely used to monitor various health conditions. However, the dynamic and heterogeneous pattern of ECG signals makes relevant feature construction and predictive model development a challenging task. In this study, we aim to develop an integrated approach for one-day-forward wellness prediction in the community-dwelling elderly using single-lead short ECG signal data via multiple-features construction and predictive model implementation. Vital signs data from the elderly were collected via station-based equipment on a daily basis. After data preprocessing, a set of features were constructed from ECG signals based on the integration of various models, including time and frequency domain analysis, a wavelet transform-based model, ensemble empirical mode decomposition (EEMD), and the refined composite multiscale sample entropy (RCMSE) model. Then, a machine learning based predictive model was established to map the l-day lagged features to wellness condition. The results showed that the approach developed in this study achieved the best performance for wellness prediction in the community-dwelling elderly. In practice, the proposed approach could be useful in the timely identification of elderly people who might have health risks, and could facilitating decision-making to take appropriate interventions.

## 1. Introduction

The accelerated growth of the elderly population in many countries and regions worldwide is creating a major burden to the healthcare system [1,2]. For instance, the existing literature indicates that the proportion of Hong Kong’s elderly will increase from 15% in 2014 to 36% in 2064 [3]. With 5% GDP in Hong Kong’s healthcare expenditures and increasing needs for elderly care, the burden of medical costs and societal resources are expected to increase significantly and rapidly. There is an urgent need to develop effective strategies for proactive and preventative healthcare.

Intelligent approaches for continuous health monitoring have the potential to promote the transition to more proactive and affordable healthcare. In the last few decades, rapid advancements in information technology, automated sensor-based data collection, and advanced data analytics methods have led to an integrated approach that supports health management of complex systems through individual monitoring and control. For example, wearable health trackers can be used to monitor gait speed, sleep time, heartbeats, daily diet, and energy consumption, etc. Most existing studies involve chronic conditions [4,5,6], rehabilitation [7,8], cardiovascular disease [9,10,11], fall [12,13,14], and general wellness [15,16,17,18].

Wellness indexes can reflect the general health status of the elderly, and are multidimensional concepts including (at least) physical, mental, social, emotional, intellectual, and environmental wellness [15,19,20]. In elderly care, the timely and accurate prediction of wellness is essential for the reduction of negative health outcomes. Traditional wellness-measuring methods are mostly self-rated questionnaires used in psychology studies as a one-time measurement instrument. These methods are not suitable for measuring wellness daily, as they are time-consuming to collect and the measure represents an individual’s health status over a long period of time.

Some research has proposed various health-monitoring systems for elderly care. For example, He et al. developed a system based on a six-layer medical cloud platform [21,22], which was mainly focused on collecting physiological signals and vital signs for generating a health assessment report. Kakria et al. developed a real-time health monitoring system for remote cardiac patients by analyzing the data from smartphone and wearable sensors [4]. Paradiso et al. presented a health monitoring system called WEALTHY for monitoring patients affected by cardiovascular disease [23]. Kailas et al. designed a general wellness platform for facilitating healthcare professionals to comprehensively track patients’ health status in real-time via smartphones [24]. The general scheme of these healthcare platforms is to first process physiological data and vital signs on-line or off-line in the back end, and then transfer the information about wellness conditions to users or healthcare providers in real-time or at a scheduled time. However, the aforementioned platforms are mainly focused on monitoring the health status of the elderly, rather than predicting a future trend. It has been well recognized that predicting health status in advance could significantly help prevent life-threatening changes from occurring and could be an effective solution to provide efficient healthcare services [25,26,27]. Yu et al. proposed a personalized healthcare-monitoring platform to forecast one-day-forward wellness conditions for the elderly [16]. By integrating vital signs data from a wearable health tracker and all-in-one station-based device, machine learning methods were adopted to predict personal wellness conditions for the elderly.

Electrocardiograms (ECGs) with their noninvasive and cost-effective merits are widely used to monitor various cardiac health conditions [28,29,30]. Conventional ECG acquisition systems such as patient monitors involve the connection of multiple skin-electrodes to the patient. Nowadays, single-lead ECG signals are conveniently available via wearable or portable health-monitoring devices without the limits of time and location. Fan et al. developed a one-day-forward forecasting method of wellness for a community-dwelling elderly population based on single-lead short ECG signals [14]. Despite the inspiring results of wellness prediction from these research, the matter of how to extract multiple useful features from the raw data for wellness prediction is still a challenge due to the dynamic characteristics of ECG signals. Different models for feature extraction have different advantages from the perspective of signal analysis. For example, the time-frequency domain features contain sophisticated information of signal patterns. Empirical mode decomposition can decompose the original signal into intrinsic mode functions (IMF) according to the order of frequency from high to low by the bandwidth of the empirical mode decomposition (EMD) filter [31]. Ensemble empirical mode decomposition (EEMD) is an improved model based on EMD which can solve mode mixing problems by adding white noise [32]. Multiscale entropy has advantages in measuring signal sequence irregularity, and is commonly adopted to reflect the self-similarity and irregularity under different scales [33]. These models for feature extraction have been successfully and widely applied in various engineering domains, but are rarely investigated in ECG signal analysis. After extracting multiple representative features, a machine learning-based model can be built for wellness prediction. Zabihi et al. proposed a framework for predicting negative health outcomes using ECG signals via random forest (RF) [34]. Athif et al. developed a method by integrating ECG signal analysis and support vector machine (SVM) for health status classification [35]. Moreover, some related research works implemented deep learning models using ECG signals. For example, Sayantan et al. adopted a deep belief network for health status prediction using ECG signals [36].

The main contributions of this article are twofold: first, multiple feature construction models were integrated to extract the hidden pattern of the ECG signal, which laid a reliable foundation for the subsequent wellness prediction. Second, a comprehensive comparative study was conducted to demonstrate the performance of the developed approach. Specifically, the original ECG signals and the features extracted from different models were cross validated with different predictive models. The experiment results show that the prediction performance of the proposed approach with feature construction strategies achieved the best wellness prediction results compared to the competitors.

The rest of this paper is organized as follows. Section 2 introduces the general procedure of the proposed approach and mechanisms of various feature-construction models. Section 3 presents the collected data sets and parameter settings prior to modeling. In Section 4, the performance of different models is systematically compared and discussed. Finally, we draw our conclusions in Section 5.

## 2. Methods

**Figure 1:**
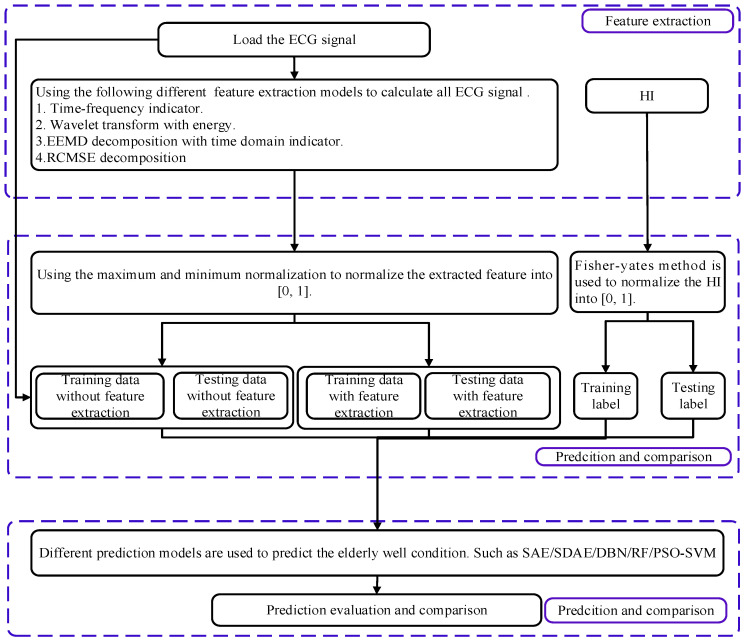
The flowchart of the developed approach (HI: health index).

The flowchart of the developed approach for wellness prediction is shown in Figure 1, containing three main stages: (1) feature construction; (2) data preparation and normalization; (3) predictive model development.

### 2.1. Features Construction Models

In this section, we present the mechanism of the four feature construction models. For a given ECG signal *X*, the following models were utilized to extract features. (1) Time– frequency domain analysis for statistical feature construction; (2) wavelet decomposition of ECG signals together with energy features calculation; (3) EEMD based model to decompose each ECG signal into a series of IMF components, and then various time domain statistical indicators were used to calculate the generated features for each IMF component; (4) refined composite multiscale sample entropy (RCMSE) models for calculating the entropy value as features from ECG signal directly. Finally, 184 features were extracted for wellness prediction. The details of four groups of features are as follows:

- Feature Nos. 1–26 (F1–F26): 26 time-frequency domain indicators were calculated, such as the absolute mean, peak, peak to peak, variance.
- Feature Nos. 27–34 (F27–F34): Using the three layers wavelet decomposition to transform the original ECG data, and then calculating the 8 energy features.
- Feature Nos. 35–164 (F35–F164): 10 IMFs were obtained from EEDM, and then 13 features from time domain were calculated by using each IMF component according to Table 1.
- Feature Nos. 165–184 (F165–F184): 20 entropy features were calculated via RCMSE.

#### 2.1.1. Time Domain and Frequency Statistical Features

Different time domain and frequency domain indicators can show different trends of a signal. For example, Tse et al. used different time–frequency domain indicators to extract a preliminary degradation trend model of the mechanical system and finally established a prediction model to realize the remaining useful life prediction [37].

To identify the representative features for describing ECG signal patterns, 13 types of time domain indicators, including absolute mean (F1), peak (F2), peak to peak (F3), variance (F4), root amplitude (F5), crest factor (F6), skewness factor (F7), kurtosis factor (F8), shape factor (F9), clearance factor (F10), impulse factor (F11), root mean square (F12), and variation coefficient (F13), were calculated, as shown in Table 1. Moreover, 13 statistical indicators extracted from the ECG signals in the frequency domain through fast Fourier transform (FFT), were also used to quantify the ECG signal. As a result, a total of 26 statistical features were extracted from each ECG signal. Let $X=\left\{x_{i}^{j}\right\}\;1\leq i\leq N\;1\leq j\leq n$ represent the given ECG signal dataset, where *N* and *n* are the length of each sample and the total number of all ECG signals.

**Table 1:** Time domain and frequency domain statistics feature. (1) *X_av_*: average value; (2) *X_p_*: peak; (3) *Xp-p*: peak to peak; (4) *Dx*: skewness factor; (5) *Xr*: root amplitude; (6) *Cf*: crest factor; (7) *SKf*: skewness factor (8) *Kf*: kurtosis; (9) *Sf*: shape factor; (10) *CLf*: clearance factor; (11) *If*: impulse factor; (12) *rms*: root mean square; (13) *K_x_*: variation coefficient.

**F1**	$x_{av}=1/N\sum_{i=1}^{N}\left|x_{i}\right|$	**F2**	$x_{p}=\max\left|x_{i}\right|$	**F3**	$x_{p-p}=\max\left(x_{i}\right)-\min\left(x_{i}\right)$
**F4**	$D_{x}=1/N\sum_{i=1}^{N}{\left(x_{i}-x_{av}\right)}^{2}$	**F5**	$x_{r}={\left(1/N\sum_{i=1}^{N}\sqrt{\left|x_{i}\right|}\right)}^{2}$	**F6**	Cf=xpxrms
**F7**	$SK_{f}=\frac{\frac{1}{N}\sum_{i=1}^{N}{\left(x_{i}-\frac{1}{N}\sum_{i=1}^{N}x_{i}\right)}^{3}}{{\left(\sqrt{\frac{1}{N}\sum_{i=1}^{N}{\left(x_{i}-\frac{1}{N}\sum_{i=1}^{N}x_{i}\right)}^{2}}\right)}^{3}}$	**F8**	$K_{f}=\frac{\frac{1}{N}\sum_{i=1}^{N}{\left(x_{i}-\frac{1}{N}\sum_{i=1}^{N}x_{i}\right)}^{4}}{{\left(\sqrt{\frac{1}{N}\sum_{i=1}^{N}{\left(x_{i}-\frac{1}{N}\sum_{i=1}^{N}x_{i}\right)}^{2}}\right)}^{4}}$	**F9**	$S_{f}=\frac{x_{rms}}{x_{av}}$
**F10**	CLf=xpxr	**F11**	$I_{f}=x_{p}/x_{av}$	**F12**	$x_{rms}=\sqrt{1/N\sum_{i=1}^{N}x_{i}^{2}}$
**F13**	Kx=Dxxav				

#### 2.1.2. Wavelet Transformation Energy Feature

The wavelet transformation can decompose the signal into various frequency bands. Hence it can reflect the changing pattern of signal in detail. The amplitude of the ECG signal can reflect the health status of the elderly in practice. For instance, the vibration amplitude of the signal will be significantly enhanced when the elderly heartbeat is faster. Therefore, calculating the energy value based on the amplitude can help effectively extract the features of the ECG signal after wavelet decomposition.

**Figure 2:**
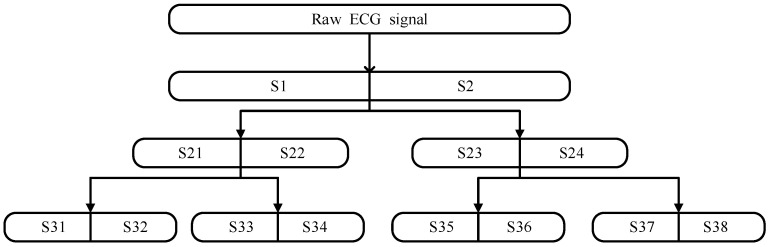
The flowchart of three layers’ wavelet decomposition.

Figure 2 shows three layers of orthogonal wavelet for the ECG signal *X*. The original ECG signal *X* is denoted as *S*. After wavelet packet decomposition, the *S*1 and *S*2 sub-bands can be obtained. After *S*1 and *S*2 are decomposed by the wavelet packet, four bands were obtained, i.e., *S*21, *S*22, *S*23, and *S*24. Eight bands of *S*31–*S*38 are obtained after the third layer of decomposition. In this paper, three layers of decomposition were used to obtain the wavelet-decomposed band. The energy $E_{w}$ of each sub-band can be expressed as,

(1) $$E_{w}={\left({\left|x_{i}^{j}\right|}^{w}\right)}^{2},\;1\leq w\leq 8$$

#### 2.1.3. EEMD with Time Domain Feature

The ECG signal makes it difficult to adaptively decompose the signal. EEMD can adaptively decompose the ECG signal according to the upper and lower extreme points. For example, Rai used EEMD to decompose the vibration signal into a series of IMF components, and then adopted the singular value decomposition (SVD) model to extract the initial degradation trend [38]. In this study, the ECG signal was decomposed into a series of IMF components in descending order.

The detailed calculation procedures of EEMD are given as follows:

(1) For the *j*th ECG signal $x_{i}^{j}$, a random white noise signal $n_{i}\left(t\right)$ is added to X(t)
  (2) $${\left(x_{i}^{j}\right)}_{p}=\left(x_{i}^{j}\right)+n_{i}\left(t\right)\;1\leq p\leq M\;$$
  where ${\left(x_{i}^{j}\right)}_{p}$ is the new signal which is added by $n_{i}\left(t\right)$, and *M* is the total number of trials.
(2) Then X(t) is decomposed into a series of IMF components through EMD by (the detailed calculation procedures of EMD is given in [31,32])
  (3) $${\left(x_{i}^{j}\right)}_{p}=\sum_{i=1}^{N_{p}}c_{ij}+r_{N_{j}}$$
  where $r_{N_{j}}$ is the residue of *j*th trial, $N_{j}$ indicators the IMFs number of the *j*th trial. $c_{ij}$ denotes the *i*th IMF of the *j*th trial.
(3) Repeat the first two steps when p<M
(4) Obtain $I=\min\left(N_{1},N_{2},\ldots ,N_{M}\right)$. and compute the ensemble means of each IMF by
  (4) $$c_{i}=\left(\sum_{j=1}^{M}c_{i,j}\right)/M\;1\leq i\leq I$$
  where $c_{i}\left(i=1,2,3,\ldots ,I\right)$ is the ensemble average of each IMF.

#### 2.1.4. Refined Composite Multiscale Sample Entropy Model

Entropy has been recognized as a useful method to measure the complexity of a time series. Pincus et al. proposed an approximate entropy (AE) method [39]. However, the AE method heavily depends on the length of the time series. As a result, the value of AE can be uniformly lower than the expected one, and lacks relative coherence especially when the data length is short. To overcome this drawback, sample entropy (SE) was developed as an improved complexity measuring method based on AE [40]. It has the advantages of high stability, low noise, interference ability, and good consistency for a large parameter range. It should be noticed that AE and SE can only reflect the irregularity of a time series on a single scale. A method called multiscale entropy (ME) was proposed to measure time sequence irregularity [33,41,42], in which the degree of self-similarity and irregularity of a time series can be reflected in different scales. Based on the multiscale sample entropy (MSE), the feature of the vibration signals can be extracted from various conditions, then the eigenvectors regarded as the input of the adaptive neuro-fuzzy inference system for roller-bearing fault recognition [43]. Moreover, the SE, in some cases, is undefined as no template vectors were matched to one another. Inaccurate or undefined SE may lead to the reduction of the reliability of the MSE algorithm. To overcome this drawback, Wu et al. [44] developed RCMSE to solve these problems and demonstrated that RCMSE can increase the accuracy of entropy estimation and reduce the probability of inducing undefined entropy.

The time series length is reduced by *τ* when the coarse-graining step shown in Equation (5) is used. For the RCMSE algorithm, the coarse-grained time series calculated at a scale factor of *τ* is given by

(5) $$RCMSE\left(x_{i}^{j},\tau ,m,r\right)=-ln\left(\frac{\bar{B^{m+1}\left(r\right)}}{\bar{B^{m}\left(r\right)}}\right)$$

where Bm+1(r)¯=1τ∑k=1τBm+1(r) and Bm(r)¯=1τ∑k=1τBm(r). Two parameters, i.e., the value of embedding dimension m and similar tolerance r of the SE should be set in advance. The *RCMSE* value can be redefined by

$$RCMSE\left(x_{i}^{j},\tau ,m,r\right)=-ln\left(\frac{\bar{B^{m+1}\left(r\right)}}{\bar{B^{m}\left(r\right)}}\right)$$

(6) =−ln(1τ∑k=1τBm+1(r)1τ∑k=1τBm(r))

$$=-ln\left(\frac{\sum_{k=1}^{\tau }B^{m+1}\left(r\right)}{\sum_{k=1}^{\tau }B^{m}\left(r\right)}\right)$$

### 2.2. Data Pretreat

The procedure of data pretreatment is divided into two parts: (1) the extracted feature values were processed by maximum and minimum normalization; (2) the elderly health index (HI) was processed by Fisher–Yates normalization and converted into output data labels.

#### 2.2.1. Maximum and Minimum Normalization

Before implementing the models to make predictions, the extracted feature set $x_{F}={\left\{x_{i}^{j}\right\}}_{F}$ was normalized into the range [0, 1]; the maximum and minimum method used for normalization is as follows:

(7) $$x_{i}^{j}=\left(x_{i}^{j}-x_{Fmin}\right)/\left(x_{Fmax}-x_{Fmin}\right)$$

where $x_{Fmax}$ and $x_{Fmin}$ are the maximum and minimum of $x_{F}$.

#### 2.2.2. Fisher–Yates Normalization

In our study, the output HI contains 5 levels (1–5) as shown in Table 2, implying the different levels of elderly wellness. Each participating elderly person was asked to self-evaluate their wellness and chose the most appropriate HI describing their current daily health conditions immediately after the assessment by health-monitoring equipment. The questionnaire was also validated in the existing literatures [16,45]. These scores of HI were self-evaluated, and thus the distribution of HI of each elderly individual varied even if their health conditions were similar. To reduce the difference in shape between a set of scores belonging to the same subject, we applied the Fisher–Yates normalization method to convert HI into 0 or 1 [16].

Assuming $HI_{ij}$ is the health index of the *j*th elder at the *i*th day, and $G_{ij}$ is the *i*th HI level of the *j*th elder, the Fisher–Yates operation of the *j*th old elder can be calculated as follows:

(8) $${\mathrm{Label}}_{j}=\;\mathrm{Fisher}–\mathrm{Yates}\left(HI_{ij}\right)=\varphi^{-1}\left(G_{ij}/I\right)\;\;1<i<I,\;1<j<J$$

where *I* is the total days of measurement data. *J* is the total number of elders.

The normalized HI value was further converted into a binary variable based on the threshold 0, where values greater than 0 representing for ‘Better’ wellness condition were categorized into class ‘0′, and the rest indicating ‘Worse’ wellness condition belong to class ‘1′. For illustration purposes, Table 3 provides an example of the normalized values of HI by Fisher–Yates normalization.

### 2.3. Prediction Methods and Performance Evaluation Criteria

After feature construction, a predictive model needs to be established for predicting the wellness of the elderly. In this study, we adopted RF, SVM with particle swarm optimization (PSO-SVM), deep belief network (DBN), stacked autoencoder (SAE), and stacked denoising-autoencoder (SDAE) to construct the predictive model, by using the extracted 184 features from the l-day lagged ECG signals and wellness outcome as the input and output, respectively, for the model development.

#### 2.3.1. Random Forest (RF)

RF is an integrated recognition classifier algorithm composed of multiple decision trees $T\left({\left(x_{i}^{j}\right)}_{F},\;\theta_{1}\right)$ 1≤l≤d, where $\theta_{1}$ is the independent and uniformly distributed random vector parameter. The classification ability of a single decision tree and the degree of correlation between each decision tree determine the generalization of the random forest algorithm. RF includes two main steps: decision tree generation and voting [46].

#### 2.3.2. Support Vector Machine with Particle Swarm Optimization (PSO-SVM)

SVM is suitable for obtaining the optimal solution in the case of limited samples [29]. SVM algorithm eventually evolved into a convex quadratic programming problem, which can avoid local extremum problems in neural networks; it can transform non-linear separable problems from low-dimensional space to high-dimensional space to achieve linear separability. In practical applications, the performance of the SVM is largely constrained by factors such as the penalty factor C and the type of the kernel function. PSO is a swarm intelligence algorithm that is widely used in parameter optimization and can effectively improve search efficiency [47,48]. In this study, we integrate SVM with PSO for parameter selection. The SVM kernel function used in this paper is the radial basis.

#### 2.3.3. Deep Belief Network (DBN)

The DBN is mainly composed and stacked by several restricted Boltzmann machine (RBM) units. Each RBM includes a visible layer *v* and a hidden layer *h*. The RBM uses the information construction output in *h* as the input of the visible layer *v*. The SoftMax classifier is used for classification at the output layer. The input layer is used to input the originally extracted features [49]. The DBN method is divided into two phases: pretraining and fine-tuning. In the pretraining phase, the data is inputted into the input layer of the DBN and connected to the first RBM for unsupervised training. After the training, the output of the previous RBM is used as the input of the next RBM until the last RBM training is completed. In the global fine-tuning phase, based on the class information of the training data, DBN performs statistics and error recognition on the categories determined by the SoftMax classifier and then uses the backpropagation algorithm to update the connection weights in DBN and reduce reconstruction error between input and output data. Therefore, the feature extracted by the last RBM is the feature extracted by DBN.

#### 2.3.4. Stacked Autoencoder (SAE) and Stacked Denoising-Autoencoder (SDAE)

Autoencoder (AE) is a kind of symmetrical three layers of the neural network [50]. The objective of AE is to reach the reconstruction error minimum and get the best output in the output layer. Several AE models can be stacked into SAE with l hidden layer. For the extracted features *X*, the total number of nodes at the input layer is the dimension of *X*. The first hidden layer is selected as the first AE to reconstruct the input data. Therefore, the input of the next hidden layer is the output of the adjacent previous hidden layer. The process is continued until the first hidden layer is trained. SAE uses the SoftMax classifier for classification at the output layer. The input layer is used to input data.

SDAE is proposed based on SAE. The main difference is that SDAE sets the input data to 0 according to the denoising probability *P* and then uses AE to reconstruct it. This enables SDAE’s robust intercourse SAE better.

#### 2.3.5. Evaluation Criteria of Wellness Prediction

The confusion matrix is a specific table layout that allows assessment of the performance of each method. A confusion matrix is given in Table 4. To evaluate the prediction performance of the different models, four criteria were used in this study, including recall (*REC*), precision (*PRE*), *F-score*, and accuracy (*ACC*). The corresponding calculation based on the confusion matrix is as follows.

*REC* = *TP*/(*TP* + *FN*) (9)

*PRE* = *TP*/(*TP* + *FP*) (10)

*F-score* = 2(*REC* × *PRE*)/(*REC* + *PRE*) (11)

*ACC* = (*TP* + *TN*)/(*TP* + *FP* + *TN* + *FN*) (12)

where *TP* is True Positive, indicating that the prediction result is positive and actually positive; *FP* is False Positive, indicating that the prediction result is positive, but actually negative; *FN* is False Negative, indicating that the prediction result is negative, but actually positive; *TN* is True Negative, indicating that the predicted result is negative and actually negative. *REC* implies the fraction of actual positives that were predicted correctly. *PRE* indicates the fraction of positive identifications that are correct. *F-score* is a harmonic mean that takes both precision and recall into consideration. *ACC* is the fraction of correctly classified cases in total.

## 3. Data Description and Model Setting

### 3.1. Subjects and Data Preprocessing

The original data were collected from a nursing home in Hong Kong. A total of 11 older adults (aged 68–86) participated in the health status assessment, including 9 females and 2 males. This study was approved by the Research Ethics Committee of City University of Hong Kong (reference number: 3-11-201709_01). The 11 individuals completed the vital signs data collection using TeleMedCare equipment (TMC, TeleMedCare Systems Pty Ltd., Sydney, Australia) under the guidance of healthcare professionals. Vital signs were collected from each elderly participant in the morning on a daily basis, and the time period of the study was three months. The data included ECG signal, systolic pressure, diastolic pressure, etc. Specifically, we extracted the ECG signal for predictive model development in this paper. The sampling frequency is 500 Hz, and the sampling time length of each ECG signal is 20–25 s. In addition, a questionnaire was assessed regularly for obtaining daily HI values.

Prior to feature construction, the original ECG signal needs to be properly segmented. In general, the larger the amount of input data, the better the feature learning performance of the prediction model. As the number of collected data samples was limited, each signal (length of 20–25 s) was segmented through a sliding window to increase the sample size of the input data. Each original ECG signal was divided into 5 s-long segments, and the sliding window was 1 s. For example, the length of an original ECG signal is 20 s, hence the ECG signal of the 1–5 s length is the first sample after being divided, and the 2–6 s is the second sample. Hence a 20-s ECG signal can be divided into 16 samples of 5 s in length. The Fisher–Yates operation was used to normalize the HI values for all samples. After preprocessing, the total number of the input sample was 6630. Among them, 3590 samples were labeled 0 and 2740 samples were labeled 1 (0 is ‘Better’ and 1 is ‘Worse’). At the model development stage, 70% of the samples with labels 0 and 1 were randomly picked for training the prediction model, and the remaining 30% of the sample was used for testing.

**Figure 3:**
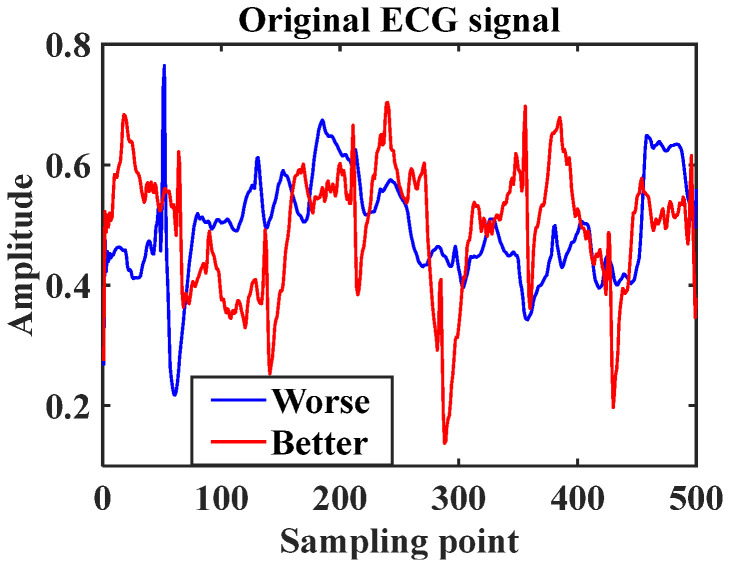
The time domain ECG signal wave of different wellness statuses.

Figure 3 shows an example of the time domain diagram of two segmented ECG signals with an output label of 0 and 1, respectively. The length of each input signal is 500. The ECG signal of ‘Better’ case had certain vibration regularity, but ‘Worse’ had a poor regularity. It worth noting that the vibration amplitude range of the two signals is relatively close, making it challenging to automatically identify the corresponding wellness status.

### 3.2. Predefined Parameters of Models

For comparison purposes, we considered both the ECG signal-based dataset without feature construction (dimension of 500) and the dataset with the extracted 184 features for modelling. The covariates of both datasets were first processed by the normalization methods, and then the converted covariates were used as input for the RF, PSO-SVM, SAE/SDAE, and DBN models to predict the wellness statuses of the elderly participants. The predefined parameters in different models were set as follows:

(1) *RF*: For the RF model development, N (N < M) features need to be selected from M variables. According to the principle of minimum node purity, one characteristic was selected from these N variables to branch growth [51]. Because M = 500 or 184, N was set as 20 and 12 in this study.
(2) *PSO-SVM*: The maximum iteration number k was set as 200 in PSO with termination tolerance *ep* = 1 × 10^−3^. The range of particle position $\left[-X_{c\max},X_{c\max}\right]$ and velocity $\left[-X_{g\max},X_{g\max}\right]$ were set as [0.1,100]. The population number was set as n=20. Additionally, the acceleration factors were set as $c_{1}=1.5$ and $c_{2}=1.7$. *r*_1_ and *r*_2_ are random numbers chosen from interval [0, 1].
(3) *SAE/SDAE/DBN*: For the SAE/SDAE/DBN model development on the original dataset without feature construction, the number of nodes at the input layer was set to 500, and the structure of the hidden layer was set to [500 500]. For the SAE/SDAE/DBN model development on the dataset with the extracted 184 features, the structure of the hidden layer was set to [184 184]. Two hidden layers were constructed with the maximum number of iterations set as 1000. The denoising rate in SDAE was set to 0.05. A sigmoid function was selected as the activation function, and the SoftMax classifier was used as the output layer. It should be noted that if the learning rate is set too large, the SAE/SDAE/DBN model easily falls into a local optimum, and the convergence rate of the SAE/SDAE/DBN model is slower [52]. In this study, the learning rate reduced linearly in 100 iterations intervals with the initial learning rate setting as 0.01.

## 4. Results and Discussion

### 4.1. Utility Analysis of Constructed Features

**Figure 4:**
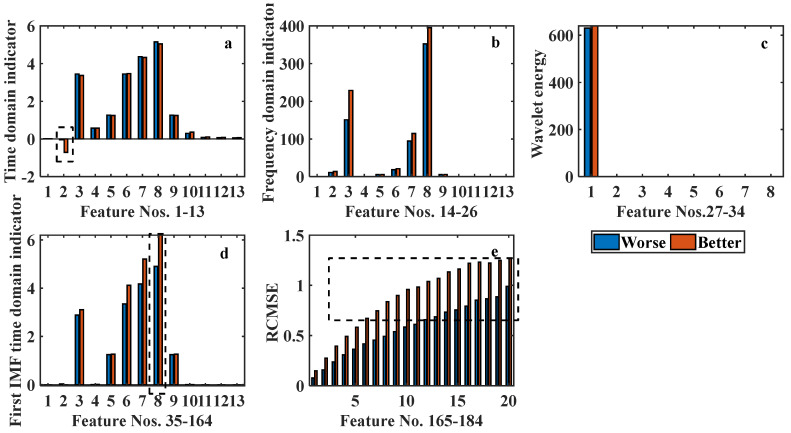
The extracted 184 features from ECG signals for one ‘Better’ and ‘Worse’ cases. (**a**) Time domain feature; (**b**) frequency domain feature; (**c**) wavelet energy feature; (**d**) EEMD with time domain feature; (**e**) RCMSE feature.

The features generated using different models presented in Section 2.1 are shown in Figure 4. Taking a comparison of ‘Better’ and ‘Worse’ wellness statuses for example, obvious differences can be observed at some representative features, as shown in the dotted rectangular area in Figure 4. Specifically, the differences between features extracted from RCMSE are the most obvious compared to the other models. This owes to the scale factor *τ* that enhances the difference. As the scale increases, the difference between ‘Better’ and ‘Worse’ cases becomes more obvious, as shown in Figure 4e. For the other feature construction approaches, the features of the ‘Better’ and ‘Worse’ cases are generally close to each other.

**Figure 5:**
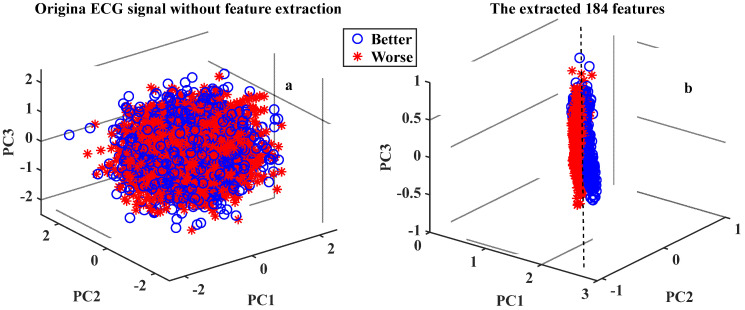
The result of the PC1-PC3 via PCA from the original ECG signals and 184 features.

To further demonstrate that the performance of the feature construction strategies in this paper are more appropriate than using the original ECG signals as input directly, principal component analysis (PCA) was applied to reduce the dimensionality of the original ECG signal without feature construction (500 dimensions) and extracted 184 features (184 dimensions) for data visualization operations. The first three principal components (PCs) were used for the 3D visualization as shown in Figure 5. As can be seen, the ‘Better’ and ‘Worse’ cases without feature construction signals are basically mixed in Figure 5a. However, the ‘Better’ and ‘Worse’ cases can be well separated by the decision boundary (black dotted line) by using the extracted 184 features in Figure 5b. These primary results lay a good foundation for subsequent prediction and improve prediction accuracy.

### 4.2. Wellness Condition Prediction and Performance Evaluation

**Figure 6:**
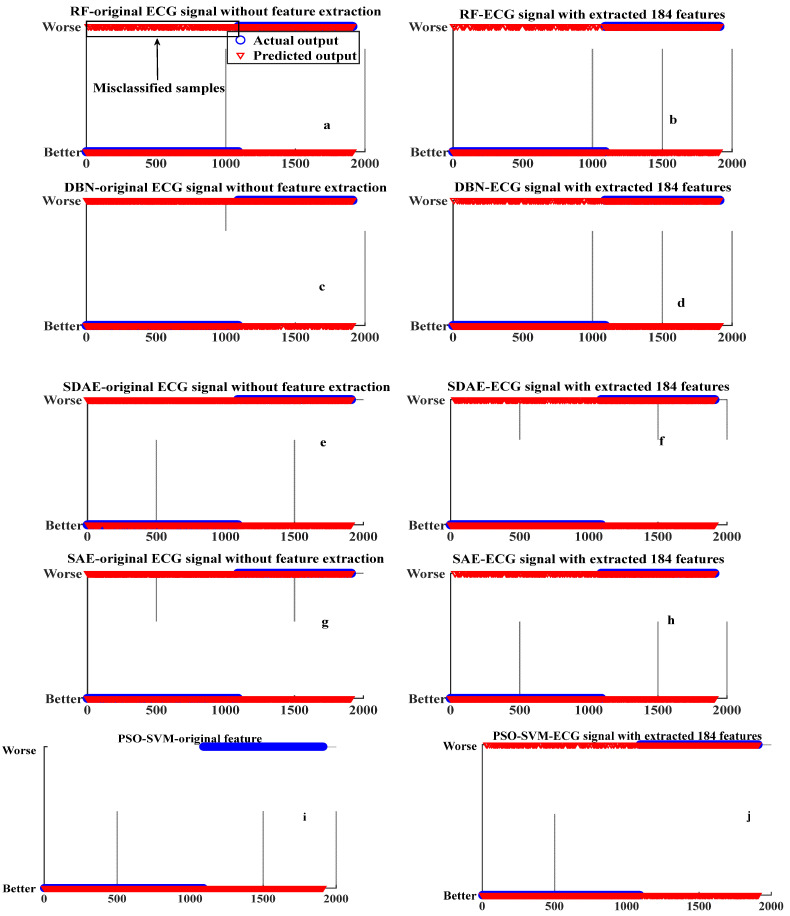
The wellness prediction result of different models.

The wellness prediction results obtained from the testing stage are shown in Figure 6, where the blue points and red points represent the predicted values and actual values, respectively. In Figure 6, the left panel shows the results from models based on the original ECG signal datasets without feature construction, while the right panel shows the results from models based on the extracted 184 features. The coincidence of the red marked sample and the blue sample indicates that the sample is correctly classified. It can be seen that the classification accuracy on the right panel is higher than the left panel. For example, the samples shown in the blocked area in Figure 6a are misclassified by RF without feature construction by using the original ECG signal.

We conducted a numerical comparison to evaluate the performance of the different models with or without the extracted 184 features. *REC*, *PRE*, *F-score*, and *ACC* were used as evaluation criteria. The corresponding results are shown in Table 5. Overall, the RF model with feature construction achieved the best prediction performance (*REC* = 0.8844, *PRE* = 0.7294, *F-score* = 0.7995, *ACC* = 79.37%) compared to the other models. The *ACC* value of all models, such as RF, PSO-SVM, SDAE, SAE, and DBN with the extracted 184 features is significantly higher than that of models without feature construction. It is worth noting that the minimum *ACC* value of the models with feature construction (68.8% for SAE) is even higher than the maximum *ACC* of models without feature construction (57.06% for PSO-SVM). The *F-score* value of all the models, including RF, PSO-SVM, SDAE, SAE, and DBN with the extracted 184 features is significantly higher than that without feature construction. The highest *F-score* is 0.7995 for RF model with feature construction. The minimum *F-score* value achieved by the models with feature construction (0.7095 for SAE) is even higher than the maximum *F-score* value achieved by the models without feature construction (0.6787 for RF). Similarly, the *REC* values of all models with the extracted 184 features are significantly higher than that without feature construction. The *REC* value of the PSO-SVM without feature construction is 1, but this was due to the highly biased results that the FN is 0 in Equation (9) for this model. In fact, the PSO-SVM without feature construction incorrectly predicted all the samples as ‘better health condition’, which is consistent with the prediction results as shown in Figure 6i. The above results imply that the feature construction method proposed in this paper is effective in improving the wellness prediction performance of different machine learning models.

### 4.3. Cross-Validation

To further demonstrate the performance of the different models with the extracted 184 features, a threefold cross-validation was conducted in this study. Thus, 6330 ECG samples containing the 3590 samples under the ‘Better’ condition (label 0) and 2740 samples under the ‘Worse’ condition (label 1) were randomly divided into three groups. A total of 67% of the ‘Better’ condition and ‘Worse’ condition samples were used as the training data, the remaining 33% samples were used as the testing data. Finally, the average value of the *ACC*, *REC*, and *F-score* were calculated for evaluating the performance of the different models. The results are summarized in in Table 6.

All evaluation indicator values of the different models with the extracted 184 features were higher than those without feature construction as shown in Table 6. Specifically, the RF model with feature construction achieved the best prediction performance (*REC* = 0.9232, *PRE* = 0.6499, *F-score* = 0.7636, *ACC* = 80.397%). Overall, the models with feature construction improved the prediction accuracy by 5–10% compared with the other prediction models. These results demonstrate that the prediction power of the model with feature construction is superior to the model without feature construction.

## 5. Conclusions

In this study, we developed an approach for a one-day-forward wellness forecast for the community-dwelling elderly via single-lead short ECG signal analysis and modeling. Based on the raw signal data, we investigated extracting, constructing, and selecting relevant features to generate critical individual health indicators correlated with different wellness conditions for subsequent analysis. After feature construction and normalization, several predictive models, including PSO-SVM, RF, SAE, SDAE, and DBN, were implemented. To demonstrate the utility of the features in enhancing prediction power, we conducted a comprehensive comparison among predictive models with and without feature construction. Our experimental results showed that the models with the extracted 184 features achieved the best performance of wellness prediction. In practice, the proposed approach could be useful in the timely identification of elderly people with increased health risks, and could facilitate the decision-making of health care providers to take appropriate interventions as needed.

In future works, we will develop feature-selection methods based on health data characteristics, with penalized methods applied simultaneously in model training. Enriched methods could give more importance to key features and reduce model overfitting by computing the parameters estimated that minimize penalized objective features, which may lead to better performance of wellness prediction. Additionally, based on the feature construction methods examined in this study, we will investigate effective approaches to integrate various data sources (e.g., PPG, Blood Pressure, and SPO2) and data types via complex big data analytics methods for a more sophisticated depiction of individual health status.

## Figures and Tables

**Table 2:** HI and wellness condition description.

HI	Wellness Condition Description
1	Poor
2	Fair
3	Good
4	Very Good
5	Excellent

**Table 3:** An illustration of output labels via Fisher–Yates normalization.

HI	3	4	5
Fisher–Yates value	−1.332	−0.627	0.449
Wellness condition	Worse	Worse	Better
Outcome label	1	1	0

**Table 4:** Confusion matrix.

	Actual (+)	Actual (−)
**Predicted (+)**	True positive (TP)	False positive (FP)
**Predicted (−)**	False Negative (FN)	True Negative (TN)

**Table 5:** The performance comparison of different models.

Model	Input Dataset	*REC*	*PRE*	*F-Score*	*ACC* (%)
RF	Original ECG signal without feature construction	0.8193	0.5792	0.6787	64.13
PSO-SVM		1	0.4997	0.6664	57.06
SAE		0.4128	0.4066	0.4097	59.32
SDAE		0.2606	0.3757	0.3077	60.37
DBN		0.3642	0.6469	0.4661	55.18
RF	The ECG signal with the extracted 184 features	0.8844	0.7294	0.7995	79.37
PSO-SVM		0.8394	0.7213	0.7759	76.96
SAE		0.8147	0.6284	0.7095	68.80
SDAE		0.8128	0.6303	0.7100	68.90
DBN		0.8450	0.6440	0.7309	71.15

**Table 6:** The performance results of different models through cross validation.

Model	Input Datasets	*REC*	*PRE*	*F-Score*	*ACC* (%)
RF	Original ECG signal without feature construction	0.8670	0.5283	0.6548	65.97
PSO-SVM		1	0.4519	0.6255	56.67
SAE		0.2110	0.3698	0.2685	61.03
SDAE		0.3593	0.4131	0.3859	58.6
DBN		0.4486	0.6257	0.5105	56.52
RF	The ECG signal with the extracted 184 features	0.9232	0.6499	0.7636	80.397
PSO-SVM		0.8822	0.6498	0.7203	78.89
SAE		0.8791	0.5491	0.6667	68.39
SDAE		0.8412	0.5688	0.6785	69.22
DBN		0.9125	0.6023	0.7255	70.25

## Data Availability

The data is available for research purpose upon reasonable request to zhaoy393@mail.sysu.edu.cn.
